# The Variety and Inscrutability of Polar Environments as a Resource of Biotechnologically Relevant Molecules

**DOI:** 10.3390/microorganisms8091422

**Published:** 2020-09-16

**Authors:** Carmen Rizzo, Angelina Lo Giudice

**Affiliations:** 1 Stazione Zoologica Anton Dohrn, Department Marine Biotechnology, National Institute of Biology, Villa Pace, Contrada Porticatello 29, 98167 Messina, Italy; 2 Institute of Polar Sciences, National Research Council (CNR-ISP), Spianata San Raineri 86, 98122 Messina, Italy; angelina.logiudice@cnr.it

**Keywords:** cold-adapted bacteria, cold-enzymes, biosurfactants, antibiotics, extracellular polymers, Antarctica, Arctic

## Abstract

The application of an ever-increasing number of methodological approaches and tools is positively contributing to the development and yield of bioprospecting procedures. In this context, cold-adapted bacteria from polar environments are becoming more and more intriguing as valuable sources of novel biomolecules, with peculiar properties to be exploited in a number of biotechnological fields. This review aims at highlighting the biotechnological potentialities of bacteria from Arctic and Antarctic habitats, both biotic and abiotic. In addition to cold-enzymes, which have been intensively analysed, relevance is given to recent advances in the search for less investigated biomolecules, such as biosurfactants, exopolysaccharides and antibiotics.

## 1. Bioprospecting in Polar Environments

According to the Italian essayist Mirco Mariucci, paradoxes contribute to the progress of human knowledge. Thus, the polar environments, lands of extremes concealing fullness of life, have established themselves as a perfect study basin in the eyes of bio-prospectors. Scientific knowledge of Poles is very scant in comparison with other areas worldwide, and a lot of aspects and sites are still available to be explored and potentially exploited. The significant uncertainty degree about what lies to be discovered beyond the austerity of the ice makes them particularly compelling for researchers. Furthermore, the paucity of in-depth knowledge on the polar biota and on the biodiversity ranges that are only at the beginning of their discovery, the pristine aspect of these environments and in the meanwhile the numberless genetic, physiological and metabolic specializations that their inhabitants have developed are the key points making these areas so attractive to researchers [1,2].

At the beginning, the polar environments appeared monochromatic and monotonous, so that it was believed they were simply desolate and lifeless lands. In reality, over the years a great diversity of environments has emerged, with unique and peculiar features, i.e., permafrost, brine, puddles, glaciers, sea. Cryo-environments are particularly harmful for microbial life from both a physical (as ice crystals and rigid temperature could damage the cellular structure) and energetic point of view (as they are characterised by low rates of mass transfer of liquid water and nutrients) [3]. What in these areas should stem and limit life, i.e., wind, dryness, low temperatures and harsh salinity conditions, stimulate it insistently, by guiding its inhabitants to develop unique adaptive strategies [4].

The main objectives in the search for novel relevant molecules are the identification of new producing species or molecular structures, with higher specificity of action. For this reason, different possible matrices for the isolation of producing bacteria have been tested. Many scientific studies on polar biota is being done with the focus of detecting new biochemical and genetic resources, with an increasing trend. In the attempt to identify unknown bioproducts or mechanisms of biosynthesis, among all the living components of polar ecosystems the microorganisms seem to be the most promising. Although the first bioprospecting investigations have been focused on the use of higher organisms as source of natural molecules, such as marine invertebrates or plant organisms, in the last decades microorganisms have been more carefully studied. They greatly have attracted the interest of researchers because they possess a series of advantages over macro-organisms that can be decisive in the bioprospecting field. Indeed, microorganisms exhibit very rapid growth rates compared to higher living beings, a factor that would favour the optimisation of production processes and replicability. Most importantly, the choice to exploit this type of bio-resource would break down the problems linked to the capture of specimens of higher organisms, which have to be preserved in order to keep the state of polar ecosystems as pristine as possible, and which in the case of Antarctic area are strongly protected by the Antarctic Treaty.

Bio-prospectors currently believe that extremophiles—assumed as microorganisms able to grow in extreme conditions of temperature, pressure, salt concentration, pH, nutrient availability—must be the focus of bioprospecting in extreme cold regions, inasmuch their biochemical processes could represent the most concrete application of polar genetic resources. According to De Pascale et al. [5], extremophilic microorganisms are essential in the search for metabolites and biocatalysts, that with their properties should reflect the extreme conditions in which they are used to live. Indeed, by living mainly in perpetually cold environments, polar microorganisms are strongly influenced at all levels, from the molecular one to the whole organisms level, and can therefore be a fundamental resource for the discovery of new cold-adapted and cold-active molecules, with endearing applications [6].

The shift of attention to the microbial world was also favoured by the development of new methodological approaches, with the advent of -omics technologies. Although always accompanied by cultivation techniques, -omics technologies have opened the doors to what is still unexplored, considering that the known bacterial species are only a small percentage of the really existing, and the unattainable, considering that only a small percentage of currently known microorganisms is also cultivable in the laboratory. Typically, bioprospecting researches avail of high-throughput screening of biodiversity or genetic materials for the discovery of new natural substances, useful for pharmaceuticals, nutraceuticals, cosmeceuticals, foodstuffs and environmental remediation [7]. In the following sections, an overview on the bioproducts from Antarctic and Arctic bacteria with biotechnological potentialities is reported. A major attention will be focused on biosurfactants, exopolysaccharides, antibiotics and enzymes.

## 2. Biotechnologically Relevant Molecules

Although bioprospecting in polar areas is as intriguing as yet unexplored issue, several researchers believe that the diversity of such extreme habitats also corresponds to the development of a great diversity of ecological niches, finally reflecting in a high degree of chemical diversity.

To date, several studies have treated a large amount and different kind of biotechnologically relevant molecules produced by bacteria isolated from abiotic and biotic matrices in cold polar environments [4,8]. The main Antarctic areas on which bioprospecting research was focused are represented by water, soil and marine sediments nearby the most active research stations, where several authors have detected a greater bacterial diversity, attributed to the influence of human activities [9,10,11]. Therefore, studies are mainly focused in the areas of King George Island (Arctowski Base), Livingston Island (Byers Peninsula) and Budd Shore (Casey Station) [12].

In the Arctic area, waters, soils and glaciers of the Svalbard Archipelago were the most exploited matrices for most of the studied biotechnogically relevant molecules, while less matrices were used as biotic resource [13,14,15].

### 2.1. Biosurfactants (BSs)

Biosurfactant are amphipathic molecules, produced by several living organisms, that act at the interface between substances at different polarity level, by exhibiting a lot of interesting actions with great specificity at peculiar conditions of pH, temperature and salinity [16]. Natural tensioactive agents are considered as a possible key to solve the problems related to the various forms of pollution both in terrestrial and marine environments. The ecological advantages and the greater functionality compared to chemical surfactants are the strengths that push the research on BS improvement [17] (Rizzo et al. 2018). The main potential application field of BSs is the bioremediation of environments contaminated with hydrocarbons and heavy metals [17,18,19,20,21]. However, they find use also in medical health and pharmaceutical areas as inhibitors of fibrin clot formation, antimicrobial, antitumoral, anti-mycoplasmic and anti-adhesive agents against several pathogenic microorganisms [22,23,24]. Recently, BSs have been suggested as a useful tool for the recovery of natural gas hydrates [2], i.e., ice lattices containing gaseous substances deriving from microbial metabolism or organic matter degradation [25], which have been detected in several areas, including permafrost sites.

Several studies focused on temperate environments have improved the actual knowledge on BS applications in the bioremediation field and suggested the exploration of new potential sources for the isolation of bacterial producers [26]. However, cold extreme environments have been rarely considered. BS are involved and included in the production of specific cell envelopes preserving the cell from salinity, temperature and osmotic pressure [27]. As recently pointed out by Perfumo and coauthors [2], the eco-friendly nature of BS is not limited only to their low toxicity and high biodegradability, but it is also correlated to the energy saving. The study of BSs from cold environment needs strong efforts, as well as BSs that remain functional at low temperature and without need for heating perfectly meets one of the main objectives of bioprospecting research, namely the reduction of production costs. Moreover, this principle could be extended also to other relevant molecules. Although data on the BS production by Arctic and Antarctic bacteria appear fragmented and scarce, the small findings treated below represent an encouraging starting point for future research. The conditions under which bacterial isolates are grown in the laboratory really often do not reflect the optimum required for the biosynthetic processes. Indeed, owing to these early studies, it has been realised that some metabolic pathways remain silent during standard cultivation conditions by avoiding the production of the molecule of interest. Recently introduced, the OSMAC (one strain many compounds) approach takes this factor into account, and aims at evidencing the production of new biomolecules from already isolated strains by introducing small variations into the cultivation conditions, in order to activate different metabolic pathways and allow the production of several biomolecules from a single strain [28]. Kristoffersen et al. [29] applied this approach in combination with a dereplication strategy on an Arctic marine *Pseudomonas* sp. isolated from halibut. By using four different cultivation media, the authors demonstrated a different bioactivity profile of extracted molecules. The isolation of four known mono-rhamnolipids, among which one rhamnolipid was novel, was achieved.

As it was suggested by available data on BS production by cold-adapted bacteria, the latest challenge aimed to increase BS productivity at industrial scale level, that is act on carbon flux by increasing the rhamnolipid precursors [30], could be intriguingly applied on cold-adapted bacteria. Another interesting development path could be also the combination of different relevant molecules, such as cold-active enzymes and BSs, taking advantage of the proven skills of some cold-adapted bacteria of concurrent capabilities [31,32].

Last but not the least, as evidenced from Table 1, which reports the list of producers considered for this review document, an important gap concerning the topic of microbial BSs is the chemical characterisation. Really often, the data provided in this regard are limited and inconsistent or relative to already known compounds, as suggested by many authors who believe that BS chemical diversity is broader than what has been described so far and many structures are still unexplored [33,34].

#### 2.1.1. BS Producers from Abiotic Matrices

Data on BS-producing bacteria are mainly derived from Antarctic areas and, at a lesser extent, from the Arctic. As it is shown in Table 1, a number of bacterial genera from abiotic Antarctic matrices have been reported as BS producers, including mainly *Pseudomonas*, *Pseudoalteromonas* and *Idiomarina* [35], *Bacillus* [34,35,36,37,38], *Rhodococcus* [35,39], *Halomonas* [40], *Pantoea* [41], *Oceanobacillus* [42], *Streptomyces* [12].

*Oceanobacillus* sp. BRI 10 from Antarctic seawater produced a glycoproteic BS in the presence of non-hydrocarbon substrates, i.e., glucose and ammonium chloride. The BS resulted stable also at high temperature and pH and did not exhibit toxicity on normal cell line [42]. The marine psychrotrophic *Halomonas* sp. ANT-3b from the Antarctic sea-ice seawater interface (Terra Nova Bay) was able to produce an emulsifying glycolipid in the presence of n-hexadecane [40]. Few additional reports are available about other marine cold-adapted BS producers from the Terra Nova Bay (Antarctica) seawater [43,44]. For example, Yakimov et al. [43] reported the production of an extracellular and cell-bound surface-active mixture of trehalose lipids that acted at the interface level with a total surface tension reduction of 40 mN/m by two Rhodococci strains. Pini et al. [44] demonstrated that *Rhodococcus* members isolated from Antarctic surface seawater samples (Terra Nova Bay, Ross Sea) employed a hydrocarbon-uptake strategy based on the BS production during growth in the presence of the sole diesel oil.

Recently, a study performed on four cold-adapted strains from Antarctic lakes and from a Cotton Glacier stream revealed two producers related to a genus, namely *Janthinobacterium,* that was not yet reported as a BS producer [45]. The two *Janthinobacterium* members, together with a *Serratia* sp. and *Psychrobacter* sp. isolates, were able to produce biosurfactants (probably sophorolipids and di-rhamnolipids as suggested by the authors) during growth at 4 °C on minimal medium supplemented with canola oil as the sole carbon source. Additionally, the production of three rhamnolipids (among which two were defined as novel molecular structures) was reported for the *Pseudomonas* BNT1 isolated from Antarctic sediments collected at 20 m in depth [46].

To date, BS production has been found to be generally associated with growth and strictly correlated to the cultivation conditions adopted. This was also observed for the Antarctic *Bacillus licheniformis* AL 1.1 from a non-contaminated sample of sand (Kroner lake, Deception Island, South Shetland Islands). A growth-associated production of extracellular BS was demonstrated for this strain with a four-fold increase of production after adjustment of media composition and physical conditions up to 860 mg/L of purified extract in 24 h [36]. The BS was identified as a lipopeptide of the lichensyn group A, D or G and it showed potentialities useful in the cosmetic industrial applications.

Malavenda et al. [35] isolated cold-adapted BS-producing bacteria from microcosms assessed with both Arctic (Kongsfjorden, Svalbard Islands, Norwegian Arctic) and Antarctic (Byers Peninsula, South Shetlands Islands) shoreline sediments. After a screening procedure, the authors selected a total of 18 BS-producing strains mainly affiliated to genus *Rhodococcus*, followed by *Pseudomonas*, *Pseudoalteromonas* and *Idiomarina*. Interestingly, in this work the authors used an integrated approach that could shed light on possible applications in the event of oil pollution, but also on the possibility of using a low-cost carbon source for bacterial BS production purposes. This aspect goes well with one of the main bioprospecting aim, namely the reduction of the production costs of the identified biomolecules. The use of sunflower oil was highlighted as an optimal low-cost alternative as after its addition to the culture medium, different *Rhodococcus* spp. strains produced BSs with better performances in terms of surface tension reduction and emulsifying activity than after the addition of tetradecane as the carbon source. Moreover, BSs produced from such cold-adapted *Rhodococcus* spp. isolates resulted functional and stable also at low temperatures (4 °C or 15 °C), by achieving E_24_ index percentages ranging from 55 to 67% and surface tension reduction up to 27.3 mN/m during incubation at 4 °C. Similarly to Malavenda et al. [35], Parhi et al. [47] (2016) reported on the Antarctic isolate *Oceanobacillus* sp. BRI10 and its BS production during growth in media supplemented with low-cost carbon and nitrogen sources. The authors indicated that the use of sugarcane juice nitrate let to a 14-fold increase of yield and a considerable decrease in the production cost, without alteration of produced BS. The genus *Rhodococcus* was often reported as a cold-adapted taxonomic group whose representatives can produce threalose lipids, a particular class of BSs with a dual cryoprotective action, i.e., preventing the water crystallization and forming a cage around the proteins to slow down the water dynamics [48].

Other studies have been performed starting from Antarctic soil samples instead of marine samples, as described above, as a direct source of new bacterial producers. Lamilla et al. [12] screened for BS production 59 bacterial strains isolated from soil samples collected in five different sites (i.e., Peninsula Byers, Fildes Bay, Robert Island, Doumer Island, and Fildes Bay-Escudero Base). Among them, *Streptomyces luridus* So3.2 produced surface biomolecules in the presence of n-hexadecane, which emulsified and displaced different oils and hydrocarbons at high levels. The authors concluded that the deriving supernatant is a possible alternative to chemical surfactants for the bioremediation of oil leakage in aquatic environments. Vollù et al. [38] explored and highlighted the biotechnological potential of Antarctic spore-forming bacterial strains that were isolated isolated from King George Island soil samples. They found BS production in strains affiliated to *Bacillus*, *Sporosarcina* and *Paenibacillus* genera, in addition to antimicrobial production and poly-enzymatic activities (see below).

Vasileva-Tonkova and Gesheva [49], by analysing the hydrocarbon oxidation ability of 17 microbial isolates from Antarctic soils (Casey Station, Dewart Island and Terra Nova Bay), detected the production of glycolipids with emulsifying activity. The same authors reported on the BS production by *Pantoea* sp. strain A-13 (deriving from Dewart Island soils) during growth in the presence of n-paraffins or kerosene as carbon sources [50].

With regard to the Arctic environment, less information are available, even if its potential as biomolecule source has been equally strengthened. A total of 130 bacterial strains deriving from Arctic soils, glaciers and rivers of the Svalbard Archipelago were screened for BS production. Among soil isolates, *Pseudomonas putida* BD2 was able to produce BSs [14]. The authors described the production of rhamnolipids in the presence of soluble substrates (e.g., glucose, molasses) and characterised the two distinct BS fractions corresponding to phosphatidylethanolamines PE(32:1), PE(33:1) and di-rhamnolipid (Rha-Rha-C10-C10). The same authors reported also on two new lipopeptide BSs, pseudofactin I and pseudofactin II, produced by *Pseudomonas fluorescens* BD5, isolated from the water of the Arctic Archipelago of Svalbard. The identified novel cyclic lipopeptides exhibited an optimal emulsification activity towards aromatic and aliphatic hydrocarbons, and several plants oils [51].

#### 2.1.2. BS Producers from Biotic Matrices

To the best of our knowledge, the potentiality of cold-adapted bacteria associated with macro-organisms have been scarcely explored for extracellular polymeric substances and antibiotics (see below), but not specifically for BSs. Only a *Pseudomonas* sp. isolated from an Atlantic halibut (*Hippoglossus hippoglossus*) in the Arctic Norwegian Sea was reported as producer of rhamnolipids, in an interesting study that evidenced the use of new useful approach in the bioprospecting-related research [29] (Table 1). 

### 2.2. Extracellular Polymeric Substances (EPSs)

Extracellular polymeric substances include intracellular and structural polymers of high molecular weight compounds with a high polysaccharidic content. Their chemical variety and arrangement have been treated by several authors [52,53], and two different forms, slime and capsular, have been described in dependence on the bond strength and adhesion to the producing cell [54]. The main activities in which their involvement has been proven are numerous, i.e., emulsifying and chelating function, or cryoprotective effect. These actions have an important ecological role in polar environments, by regulating a lot of processes that are more decisive for survival in unfavourable environmental conditions such as the polar ones, i.e., the cellular aggregation processes, the biofilm formation, the nutrients and trace element uptake as well as the preservation from desiccation [55]. Indeed, they are generally produced by microorganisms to cope with harsh environmental conditions, as a defence strategy against possible contaminants, or to facilitate nutrient intake.

Differently from BSs, the research for EPS production from extremophiles is a little bit richer, and involves microorganisms isolated from both polar environments [56,57], even if great part of cold-adapted bacteria able to produce such compounds have been isolated from abiotic matrices [56,57,58,59,60,61,62]. The most reported genera of cold-adapted EPS producers include *Pseudoalteromonas* and *Halomonas* [59,60,61,62,63]. The selection of data used for the present text on cold EPS bacterial producers are reported in Table 2.

#### 2.2.1. EPS Producers from Abiotic Matrices

Studies on both Arctic and Antarctic sea-ice communities highlighted the important role of bacterial EPS production for the organic carbon balance in the sea and ice-water interface [64].

Indeed, Antarctic seawater and sea-ice are considered promising sources as well as the ice formation processes from seawater led to the establishment of highly variable microenvironments with peculiar conditions of temperature, salinity, nutrient concentration and irradiation [62,63], which are ideal for EPS production. This was suggested by several studies, as it is the case of the increased EPS production reported by Mancuso Nichols et al. [61,62] for *Pseudoalteromonas* spp. strains isolated from Antarctic seawater and ice samples of the Southern Ocean. The productivity of the isolate during growth at 2 and 10 °C resulted three times higher than at 20 °C, and the temperature also affected the chemical structure of EPSs, with a higher uronic acid content that was recorded.

The same site was useful for the isolation of ten bacterial EPS producers by the same research group [62], including representatives of *Pseudoalteromonas*, *Shewanella*, *Polaribacter*, and *Flavobacterium* genera. The authors highlighted the presence of uronic acids and sulphates, but also a strong chemical difference between EPSs, even among the six *Pseudoalteromonas* isolates. This confirms the importance of investigation in extreme environments, which could harbour a very highly diversified microbial community, with a resulting high level of chemical diversity. An optimisation procedure was carried out by Caruso and coauthors [55], in order to establish the optimal conditions for EPS production by the Antarctic strain *Pseudoalteromonas* sp. MER144 isolated from seawater (Terra Nova Bay, Ross Sea). The bacterial isolate resulted able to produce a higher amount of EPSs during incubation at 4 °C and pH 7, with addition of 2% sucrose (*w*/*v*) and 3% NaCl (*w*/*v*). Moreover, the biosynthesis processes were stimulated by the addition of heavy metals to the culture media. A similar procedure was applied on a *Marinobacter* sp. W1-16 isolated from Antarctic surface seawater, who produced a 260 kDa EPS (optimal conditions 15 °C, pH 8, 2% glucose (*w*/*v*), 3% NaCl (*w*/*v*) [65] (Caruso et al. 2019). The chemical analysis evidenced a higher quantity of carbohydrate than of proteins and uronic acids, as well as the presence of sulphate, and several biotechnological properties were demonstrated, namely emulsifying activity, cryoprotection, heavy metal bindin.

*Pseudoalteromonas haloplanktis* TAC 125 strain from Antarctic seawater was able to produce lipo-oligosaccharide and exopolysaccharide components [59]. The EPSs composition disclosed the presence of proteins (40%) and carbohydrates (10%), and a higher phosphate content at higher incubation temperature. Interestingly, this finding allowed the authors to confirm the importance of temperature as parameter affecting the biosynthesis processes, and to suppose a direct effect on kinase activity.

Antarctic marine sediments, which could represent an even more hostile and peculiar environment able to stimulate the development of unusual characteristics and functional activities in their bacterial inhabitants, have been also explored for the search of BS-producing bacteria. Carriòn and coauthors [58] isolated a *Pseudomonas* sp. ID1 strain from South Shetland Islands (Antarctica) with highly mucous colonies and characterised an EPS composed of glucose, galactose and fucose with a molecular mass over 2 × 10^6^ Da. Similarly, Kim et al. [60], isolated twenty-five strains from sediment of King George Island (Antarctica), among which the *Pseudoalteromonas* sp. KOPRI 21,653 exhibited the production of an EPS containing galactose and glucose. A study on the biotechnological potential of cultivable bacteria from brine lenses of three Antarctic lakes (located in the Boulder Clay and Tarn Flat areas) provided interesting insights in the bioprospecting field. The 19.5% of total isolates showed mucoid aspect, and four isolates (namely *Pseudomonas* spp. BC1-139 and BC1bis-18 from Boulder Clay, and *Psychrobacter* TF4-72 and *Pseudomonas* TF5-192A from Tarn Flat) produced promising amounts of EPS (from a minimum of 20.5 to a maximum of 170.1 μg EPS mL^−1^) [66].

The Arctic resources have been less exploited than Antarctic ones, and few reports are available. Sathiyanarayanan et al. [67] reported on the screening of 53 Arctic bacteria from glacier soils and the subsequent selection of a novel *Flavobacterium* sp. ASB 3-3 as EPS producer. Interestingly, the chemical characterisation of the EPS revealed a peculiar composition, based on the presence of D-glucose and D-galactose repeating units, but mannose free, which is a common constituent of Antarctic and Arctic bacterial EPSs. A *Pseudoalteromonas* sp. strain was reported as an optimal EPS producer among a total of 110 screened bacteria isolated from Artic sea-ice [68]. The EPS revealed a complex structure of α-mannan of a molecular mass superior than 2 × 10^6^ Da. Marx et al. [69], reported the production of a cryoprotectant EPS by a *Colwellia psychrerythraea* strain 34H, isolated from Arctic marine sediments, and demonstrated the strong influence of extreme conditions on the biosynthesis processes, by noting that harsh temperature, pressure and salinity stimulated the EPS production from the strain. Finally, an EPS composed of mannose and galacturonic acid (ratio 3.3:1.0) and molecular weight of 1.7 × 10^7^ Da was characterised by Kim et al. (2016) as a product of the *Pseudoalteromonas* ArcPo 1 strain isolated from the Chukchi Sea in the Arctic Ocean [70].

#### 2.2.2. EPS Producers from Biotic Matrices

The use of biota, mainly sponges, as a source for the isolation for bacterial EPS producers has been only recently reported for polar environments.

Recently, Caruso et al. [71] reported the EPS production by cold-adapted bacteria isolated from Antarctic sponges (Terra Nova Bay, Ross Sea). Four sponge-associated Antarctic bacteria (namely *Winogradskyella* sp. strains CAL384 and CAL396 from *Tedania charcoti*, *Colwellia* sp. strain GW185 from *Hemigellius pilosus*, and *Shewanella* sp. strain CAL606 from *Haliclonissa verrucosa*) were selected among 1583 isolates as they produced extracellular polymeric substances with a moderate content of carbohydrates (with galactose, glucose, galactosamine and mannose as the principal constituents), protein and uronic acids. The authors explored also the biotechnological potential of these EPSs. The strains were more efficient during incubation at a suboptimal incubation temperature (4 °C), thus suggesting a probable biosynthesis in response to stressful conditions. To the best of our knowledge, bacteria associated with marine invertebrates from polar environments were not further investigated. The potential of sponges as useful source of strains possessing biotechnological values was also recently evidenced through metagenomic approaches. 

### 2.3. Antibiotics (Abs)

The interest in molecules with antibacterial activity increased in the past decades because of the increment of resistant bacteria to commonly used antibiotics [72]. This represents an urgent problem in many fields, not only for the human health but also for the management of aquaculture systems. The search for new natural compounds that can replace the commonly used antibiotics has spread to different areas, and it is open to consider several possible sources, including polar matrices. The main sources for drug discovery have been represented for a long time from terrestrial bacteria and fungi or higher plants, but in a next step also marine microorganisms have started to be considered and proven as natural product producers [73]. Among the several effects that certainly the extreme polar conditions exert on microorganisms there is the development of defence strategies, often translatable in the production of metabolites with antimicrobial activity. Several authors assessed the importance of cold-adapted bacteria as potential new source of compounds useful for the control of pathogenic microorganisms [74,75,76,77,78], owing to their typical survival strategies, such as antagonistic activity, or sophisticated communication mechanisms that could imply the production of special defensive metabolites [79,80]. In some cases, strains of polar origin investigated for their enzymatic potential showed also antibacterial strains against a number of pathogens (see below and in Table 3).

Antarctic culturable bacteria with antimicrobial activity are mainly affiliated to Actinobacteria, Gammaproteobacteria, Firmicutes and Cyanobacteria [74,76,81,82,83,84,85,86,87,88,89] (Table 3). Antagonistic properties of microorganisms inhabiting extreme environments have been also investigated [90], but it was not extensively improved as for mesophiles [74,76], and the traditional culturable techniques applied to underexplored environments still represents a potential productive basin.

#### 2.3.1. Abs Producers from Abiotic Matrices

The most exploited abiotic sources in Antarctica are represented by sediments and soils, but also seawater and sea-ice have been investigated as suitable matrices. The culturable microbiota associated with polar ice is dominated by Alphaproteobacteria members, such as *Octadecabacter* [91,92], and Gammaproteobacteria and Bacteroidetes among which *Glaciecola* and *Salegentibacter* are reported as producers of bioactive natural compounds [93,94]. As regards the Arctic area, seawater is dominated by *Roseobacter* clade members, Gammaproteobacteria, and Actinobacteria affiliates, among which numerous groups show antagonistic traits [95,96,97,98,99].

A screening carried out on a panel of 63 cold-adapted bacterial strains isolated from Antarctic seawater of South Shetland and Deception islands revealed three *Halomonas titanicae* affiliates able to produce low-molecular weight antimicrobials with stability in wide pH and temperature ranges. The isolates showed a wide inhibition spectrum against both human pathogenic and phytopathogenic bacteria (i.e., *Salmonella* spp., *Escherichia coli*, *Enterobacter aerogenes*, *Serratia marscecens*, *Shigella* spp., *Staphylococcus* spp., *Xanthomonas* and *Erwinia*) [100].

Twenty-four Antarctic bacteria isolated from sediment and soil samples from Deception and Galindez Islands were recently screened for their antimicrobial activity by Tomova et al. [101], by showing the inhibition of at least one of the eight indicator bacteria. Interestingly, some of the isolates were able to inhibit the known human pathogenic bacteria, i.e., *Escherichia coli*, *Pseudomonas aeruginosa* and *Acinetobacter johnsonii* [101]. Among the isolates, the strain *Pseudomonas* sp. A1-1 exhibited the broadest inhibitory spectrum, by resulting active against all target bacteria and yeast cultures. An Antarctic soil sample isolate, *Janthinobacterium* sp. SMN 33.6, was reported as able to possess antibacterial activity against different strains, namely *Serratia marcescens*, *Escherichia coli* and *Acinetobacter baumannii* and *Pseudomonas aeruginosa*, with MIC values ranging from 0.5 to 16 μg mL^−1^ [88]. Antibacterial activity from *Streptomyces* spp. strains isolated from Antarctic soil samples was evidenced by several authors [102,103,104] against both seven Gram-negative and eight Gram-positive pathogens. A broad spectrum of antibacterial activity was demonstrated for the strains affiliated to this taxonomic group, and the genome sequence analysis revealed a large strain-level diversity in biosynthetic genic clusters, of which only a fraction is expressed in laboratory conditions. Other Actinobacteria members living in Antarctic environments have been proven to be rich sources of antibacterial metabolites, as in the case of Actinobacteria from volcanic soil at Deception Island, including *Gordonia*, *Leifsonia* and *Terrabacter* affiliates [105], and those from soils of Barrientos Island, among which *Brevibacterium* affiliates showed the highest and broadest antibacterial activity [106].

The antagonistic activity from Antarctic bacteria is exhibited against different targets and could be specie-specific. Mojib et al. [107] proved antimycobacterial activity for two pigments isolated from bacterial strains (*Janthinobacterium* sp. Ant5-2 and *Flavobacterium* sp. Ant342 producing violacein and flexirubin, a violet and a yellow-orange pigment, respectively) of the freshwater lakes of Schirmacher Oasis, East Antarctica. Similarly, antifungal activity against plant pathogenic fungi was evidenced for *Bacillus* sp. Pc3 isolated from Antarctic seawater, whose genome was fully sequenced [108]. Wong et al. [109] reported the antagonistic activity of the strains *Pedobacter cryoconitis* BG5, *Pseudomonas migulae* WEK1, *P. corrugata* WEA1 and *Pseudomonas* spp. MTC3, MA2, CG21 against several foodborne pathogens. Similarly, *Bacillus*, *Rummeliibacillus*, *Paenibacillus* and *Sporosarcina* members isolates from the same site (King George Island) inhibited the growth of *Staphylococcus aureus* and *Candida albicans* was detected [36]. In addition to antimicrobial activity of *Arthrobacter*, *Psychrobacter* and *Rhodococcus* isolates also showed antiproliferative and antiparasitic activities [110].

Recent investigations have allowed to detect and improve the knowledge about volatile bioactive compounds (VOCs) with activity against *Burkholderia cepacia* complex (Bcc) strains, first determined by Papaleo et al. [72] for sponge-associated bacteria (see below) and then evidenced also for the Antarctic seawater *Pseudoalteromonas haloplanktis* TAC125 [111].

The activity against Bcc strains was proven also for cold-adapted strains isolated from Antarctic sediments collected at -20 m of depth, affiliated to *Psychrobacter*, *Pseudomonas* and *Arthrobacter* [46]. Among them, the rhamnolipids produced by *Pseudomonas* BNT1 were first reported as antagonist agents of Bcc strains. Sannino et al. [112] demonstrated the role of methionine addition to the growth medium on the antagonistic activity of *Pseudoalteromonas haloplanktis* TAC125 and how methylamine contributes to the inhibitory action. The same strain was reported for its antibiofilm activity against the biofilm-producing *Staphylococcus epidermidis* [113]. Antibiofilm activity was demonstrated also from a series of cold-adapted bacteria isolated from the Fildes Península against *Flavobacterium psychrophilum* 19749, among which *Pseudomonas* sp. M19B was demonstrated as the most efficient [114].

Arctic sources have been recently exploited by Zhang et al. [115], who published an interesting article on the antibacterial activity of silver nanoparticles produced by the Arctic anti-oxidative bacterium *Paracoccus* sp. Arc7-R13. Among the metal nanopartcles, silver nanoparticles have gained great interests because of their intriguing applications in numerous fields (i.e., biomedicine, agriculture, medicine) owing to their catalytic properties as well as biological effects, including their use as potential bactericidal agents against pathogenic bacteria [116]. Zhang and coauthors [115], in addition to proving that such peculiar particles were active against *Bacillus subtilis*, *Staphylococcus aureus*, *Pseudomonas aeruginosa*, *Escherichia coli*, reported an advantageous method for obtaining silver nanoparticles by using the bacterial supernatant. Indeed, the study reveals that many compounds dissolved in the supernatant are involved in the formation of these metal nanoparticles, thus proposing an innovative and underexplored approach. A recent study reported the isolation of two diketopiperazines 1 and 2, two phenazine alkaloids 3 and 4, and an indole carbaldehyde 5 and of a benzoin acid derivative from an Arctic *Pseudomonas aeruginosa* strain, isolated from seawater of Arctic Chuckchi Sea [117]. The authors elucidated the chemical structure of the single fractions and proved an antibacterial action against *Staphylococcus aureus* and *Candida albicans*. The inhibitory action was high for compound 1, which exhibited IC_50_ value of 7.17 μM against *S. aureus*, and IC_50_ value of 20.03 μM against *C. albicans*. An uncommon bacterial genus was recently reported for antibacterial activity by Rizzo et al. [118], namely two Arctic *Salinibacterium* spp. strains previously isolated from the Kongsfjorden (Svalbard Islands, High Arctic Norway) [119]. Indeed, the concentrated supernatants obtained resulted active against *P. damselae* subsp. *piscicida*, a relevant pathogen in aquaculture field.

Wietz et al. [120] studied the antibiotic-producing Arctic strains from the central Arctic Ocean by and found seven related *Arthrobacter* spp. strains as producers of arthrobacilins A to C under different culture conditions and observed two potential novel analogues. The antagonistic activity was exhibited against a number of Gram negative and Gram positive bacterial pathogens. Despite *Arthrobacter* genus includes many known terrestrial strains with antagonistic activities, less is known about co-generes from polar environments. The isolation of antagonistic *Arthrobacter* strains from distant habitats led the authors to suppose a broad niche-specificity and a wide distribution.

Special Arctic habitats have been recently reported as untapped source of novel antibacterial molecules. This is the case of Marcolefas et al. [121] who evidenced antibiotic activity against foodborne and clinical pathogens by bacteria isolated from Arctic permafrost, saline spring sediments and cryptoendoliths. Specifically, two promising strains were retrieved, namely *Paenibacillus* sp. GHS.8.NWYW.5 and *Pseudomonas* sp. AALPS.10.MNAAK.13, and through proper genome sequencing and mining specific gene clusters involved in the synthesis of putative secondary metabolite. Moreover, the low homology level detected in comparison with genic clusters previous identified within the genome of the same species suggests the potential production of unknown compounds and promotes the exploration of unused resources.

#### 2.3.2. Abs Producers from Biotic Matrices

The health and medical fields represent the first that started to consider marine organisms as optimal source for new natural bioactive compounds. In 1996, Jayatilake and coauthors [122] wrote about the importance of exploring biological matrixes to obtain bacterial symbionts able to produce bioactive molecules. Among the microorganisms associated to the Antarctic sponge *Isodictya setifera*, a *Pseudomonas aeruginosa* isolate allow to detect several molecules, namely new natural products and phenazine alkaloid antibiotics, which were active against Gram-positive microorganisms.

A number of works [123,124,125] reported the use of Antarctic benthic microbial mat as a source of antimicrobial producers, and found several members of Cyanobacteria, together with two Gammaproteobacteria members (*Psychrobacter* sp. and *Shewanella* sp.) and a Betaproteobacteria (*Janthinobacterium* sp.) as promising producers.

Mangano et al. [90] reported about antagonistic interactions among cultivable bacteria isolated from the Antarctic sponges *Anoxycalyx joubini* and *Lissodendoryx nobilis*. The study found that these types of interactions, between bacteria associated to the same sponge species and bacteria associated to different sponge species, could have an important role in shaping the bacterial communities within their hosts. Moreover, Mangano and coauthors highlighted the strong potential of Antarctic bacteria for their antibacterial activity and suggested their biotechnological potential. Papaleo at al. [72] used the same biological matrix for the isolation of new bacterial producers of antimicrobial compounds, and interestingly focused the study on the effect against cystic fibrosis opportunistic pathogens belonging to the *Burkholderia cepacia* complex (Bcc). Also, in this case the sponge-associated bacteria were proposed as optimal potential producers of bioactive molecules, and 140 bacterial strains were isolated from three Antarctic sponge species, namely *Haliclonissa verrucosa*, *Anoxycalyx joubini* and *Lissodendoryx nobilis*, collected from the area of Terra Nova Bay coast (Ross Sea). The results allowed to detect volatile organic compounds (VOCs) that exhibited a specific inhibition action towards *Burkholderia cepacia*, without inhibitory effect on other pathogenic bacteria. Moreover, the volatile compound presented a higher effectiveness against Bcc bacteria than common antibiotics, such as ampicillin, tetracycline, rifampicine, chloramphenicol, ciprofloxacine, gentamicin, nalidixic acid. The authors suggested that the occurrence of symbiotic relationships between bacteria and marine invertebrates, sponges in this specific case, could strongly stimulate the production of molecules with antagonistic activity, necessary to maintain an ecological balance in the bacterial populations. Moreover, they detect a strong specificity of bacterial taxonomical groups with the sponge species, also correlated to the production of antimicrobial compounds able to inhibit antagonistic bacteria, as previously suggested by Mangano et al. [90]. Those investigations were improved by more in-depth analyses on specific strains. Indeed, Papaleo and coauthors [111] investigated the volatile profile under aerobic conditions, and the potential influence of the growth medium for the three Antarctic sponge strains *Pseudoalteromonas haloplanktis* TB41, *Psychrobacter* sp. TB67 and TB47 to which the seawater strain *Pseudoalteromonas haloplanktis* TAC125 was added. The authors found that *Pseudoalteromonas* strains were more effective than *Psychrobacter* strains and suggested that differences in antagonistic activity have to be attributed to the taxonomical position rather than to their isolation site, by considering the different origin of the strains.

### 2.4. Cold-Enzymes

Psychrophilic enzymes are molecules of great concern and importance for the adaptability of microorganisms in polar environments. Differently from the other relevant molecules described in the above sections, the enzymatic activities of cold-adapted bacteria have been extensively investigated in polar areas. A large amount of different cold enzymes has been identified [126], most of them being suitable in industrial applications, but not enough convenient to meet all requirements of industry.

Cold enzymes possess high catalytic efficiency at low temperature and great molecular flexibility, suitable features that make them really attracting for the industrial and biotechnological application in a sustainable and not expensive way [4]. The increased flexibility is generally due to a decreased number of hydrogen bonds and salt bridges [127,128]. Among the several adaptations in cold-adapted enzymes, the prevalent structural feature is the higher surface hydrophobicity level and negative charge, owing to the higher Glu+Asp/Arg+Lys ratio than the mesophilic enzymes, as revealed by the study of crystal structure of 11 proteins isolated from the *Oleispira antarctica* strain [129]. Moreover, a reduced proline and arginine content led to a higher molecular entropy, while the increased accessibility of active site and lower interactions between subunits and domains provide a greater flexibility for substrate and cofactor binding. Despite a number of common features have been observed among cold-enzymes, it must be emphasised that in some cases a small number of specific residues have proven as uniquely responsible of the cold adaptation. The cold life-style is not only associated to alterations in individual enzymes, but it is often the result of gene regulation processes and specific pathways activation [130].

The main obstacles to the concrete use of these compounds in the industrial field are their thermolability, high costs of production and processing at low temperatures [131]. Despite this, the topic remains amazing in the eyes of researchers, who currently care about energy saving attitude and prevention of undesirable chemical side reactions [4]. According to Sarmiento et al. [132], the use of cold-enzymes brings numerous advantages, by reducing contamination risk and the release of chemical by-products which could occur at high temperature during the production processes in food industry. From an ecological point of view, enzyme-producing bacteria play a crucial role in the organic carbon and nutrient metabolism in Antarctic and Arctic marine environments, especially proteases, considering that proteins are the main components of sedimentary marine POM [133,134]. Moreover, the bacterial fractions with strong enzymatic abilities seem to be more successfully adaptable to the changing environment that characterises polar areas [135].

To date, the synthesis of cold enzymes by psychrophilic strains has been more improved for bacteria isolated from abiotic matrices [136,137]. Many environments have been considered as suitable sources, such as Antarctic soil and sediments [138,139], Arctic and Subarctic glaciers [140,141,142,143], deep sea, permafrost soils and active layer [144,145,146] (Table 4). Most studies are focused on a small number of microbial species and they do not improve all the aspects, such as the optimal functioning temperature or the chemical structures, thus leaving mostly unknown the diversity of polar microbes with potential for cold enzymes and the optimal conditions for the enzyme working.

#### 2.4.1. Cold-Enzyme Producers from Abiotic Matrices

First reports on cold enzymes detected and characterised several kinds of molecules and functions, such as α-amylase from the seawater strain *Alteromonas haloplanktis* [147,148,149], subtilisin from *Bacillus* sp. TA39 and TA41 [150,151,152], lipases from Antarctic *Psychrobacter immobilis* BI0 and *Moraxella* TAI44 [153,154,155,156].

More recently, Fukuda et al. [157] discovered esterase, amylase and protease production in *Lysobacter oligotrophicus* isolated from an Antarctic freshwater lake. Proteases are generally the most attracting enzymes for industrial purposes, covering several fields, such as detergent, textile and food industry, bioremediation and biocatalysts under low water conditions [158]. Protease production has been detected for the strains *Sporosarcina aquimarina* and *Algoriphagus antarcticus* isolated from Antarctic soil of King George Island. The two proteases, of 55 kDa and 90 kDa exhibited best activity at 27 °C and 37 °C [159]. The proteolytic zymograms suggested their identification as metalloproteases as the only one, with best activity at pH values of 5.0–7.0 for *S. aquimarina* and 7.0–9.0 for *A. antarcticus*.

A total of 71 microbial strains isolated from Antarctic freshwater lakes showed proteolytic activity at 4 °C and presented a highly diversified affiliation among the three lakes, including *Flavobacterium*, *Pseudomonas*, *Arthrobacter*, *Psychrobacter*, *Cryobacterium*, *Hymenobacter* and *Polaromonas* affiliates [160]. The highest activity was evidenced for the protease produced by *Pseudomonas prosekii* strain ANS4-1, and proteases from four representatives among total isolates maintained the 30% of activity at 0 °C. According to the authors, all proteases were classified as metalloproteases, with the only exception of the serine protease secreted by *P. cryohalolentis* strain ANH4-1. Similarly, metalloproteases with optimal activity at 40 °C and pH 7–9 were described for *Pseudomonas* spp. strains [133] and at 45 °C and pH 6–10 for *Pseudoalteromonas* sp. strain P96–47 [161]. *Bacillus* spp. were detected also as producers of extracellular proteases, with optimum activity at 40 °C and pH 7.4 (i.e., proteases from *Bacillus* sp. JSP1 with great efficiency in casein, keratin, and gluten hydrolysis) [162]. This value is similar to the optimum temperature (35 °C) detected for the protease secreted by *Colwellia* sp. NJ341 isolated from Antarctic sea ice [163]. JSP1 protease was characterised as a neutral protease belonging to the metalloprotease class, able to hydrolyse more efficiently the casein, but with also a regardable activity on keratine and gluten. The enzyme was also active with gelatin, collagen, bovine serum albumin, L-leu-p-nitroanilide and N-succinyl-L-phe-p-nitroanilide, by demonstrating the strong potential for application as environmentally friendly feed additive and in poultry and leather industries, in terms of broad substrate specificity and ph and temperature range functioning. Differently, the purified protease from *Colwellia* sp. NJ341 was inhibited by phenylmethylsulfonyl fluoride, suggesting that it is a serine protease. By SDS-PAGE and MALDI-TOF MS resulted a molecular mass of 60 kDa, and it was active from pH 5–12 and also at 0 °C at the 30% of the maximum activity extent.

In addition to protease, a conspicuous poly-enzymatic activity was detected for strains isolated from sediment and soil samples of Deception and Galindez Islands, with higher potential for the former. Ureases, polygalacturonases, β-glucosidases, phytases and ribonucleases were detected at different extent among phylogenetic groups, but with a first report for the polygalacturonase production by Antarctic bacteria and β-glucosidase production by culturable Antarctic *Burkholderia* strain [164].

A really interesting finding was also the production of a cold-active iron superoxide dismutase (SOD) by an Antarctic sea ice isolate, *Marinomonas* sp. NJ522. The purified SOD showed a molecular mass of 48 kDa with highest activity at pH 8–10 and temperature 40 °C and maintained a 35% of the maximum activity at 0 °C [165]. As it was pointed out by the authors, this result has important implications in medical and cosmetic fields, as the SOD production was rarely reported for cold-adapted bacteria and it works at temperature ranges near human body physiological temperature.

Multi-enzymatic activities were more easily retrieved in studies performed on larger collection of bacteria. Gratia et al. [166] performed a screening procedure in more than 1000 psychrophilic strains, isolated from different cold environments, and proved the production of at least two kinds of enzymes for each strain, among proteases, lipases, amylases, cellulases and xylanases during incubation at 4 °C. Tropeano et al. [135] applied an interesting approach on a collection of bacteria isolated from different matrices (water and sediments, but also biotic sources, *see below*) of Potter Cove, Antarctica. After detection of protease-producing cold-adapted strains, they adopted a screening procedure that allowed them to identify multiple-enzyme producers. The study evidenced the production of pectinases, cellulases, xylanases, amylases and agarases, and revealed the great potential of *Pseudoalteromonas* isolates for the cold-enzymes bioprospection and the relevance to the cycling organic matter, in line with previous reports [167,168,169,170,171]. The production of cold-enzyme classes different from proteases are equally important for the metabolism of POM components in aquatic environments [134,172,173]. Similarly, a total of 518 Antarctic microorganisms deriving from different matrices (air, ice, sea and freshwater, soil, sediment, bird and marine animal faeces, dead animals, rocks and algae, plants, microbial mats as biotic sources, *see below*) and including also yeasts and filamentous fungi in addition to bacteria were studied for enzymatic activities [174]. *Pseudomonas* spp., *Psychrobacter* sp., *Arthrobacter* spp., *Bacillus* sp. and *Carnobacterium* sp. resulted good producers of amylase, lipase, gelatinase, caseinase and protease, with some bacterial clones that were able to produce also ligninase, xylanase and cellulase. The authors interestingly correlated the specific enzymatic activities with the origin of strains, as in the case of the highest amylase activities exhibited by *Arthrobacter* spp. strains, recovered from sediments and pieces of wood (*please see below for biotic sources of isolation)*.

Various members of Actinobacteria group were pointed out also by Lamilla et al. [175], in a study focused on 30 culturable Actinobacteria samples from the South Shetland Islands, Antarctica. Indeed, the taxonomic groups retrieved were *Arthrobacter*, *Brevibacterium*, *Curtobacterium*, *Janibacter*, *Knoellia*, *Rhodococcus*, *Streptomyces* and *Thermoleophilum*, and isolates showed production of at least one extracellular enzyme at 4 °C with protease, gelatinase and cellulase most common. Interestingly, the authors observed that proteolytic activity was exhibited with particular extent by sediment bacteria (mainly in Hannah Point, Armonía Point and Fildes Bay), while greater amylolytic and cellulolytic activities were detected in bacterial isolates from sediments. Lipolytic activity was instead detected as a characteristic common to all strains. As showed for BS production, Vollù et al. [38] reported also a good potential for enzymatic activities (esterase, caseinase, amylase and gelatinase for 45%, 30%, 16.2% and 15% respectively out of a total of 80 isolates) by aerobic endospore-forming Antarctic bacteria.

Several authors [176,177] suggested the dependence of isolation temperature on the proteases and, more generally, on cold-enzyme (i.e., cellulase and pectate lyase) characteristics and functions, without any dependence on the taxonomic affiliation of producer nor the chemical nature of enzyme. These findings therefore highlight the importance of the screening strategies chosen for detecting psychrotolerant bacteria able to produce strong or weak cold adapted enzymes. Contrastingly, Olivera et al. [178] characterised proteases and thermokinesis of different affiliated bacteria from sediment samples from subantarctic areas in Argentina, by suggesting important variations related to the bacterial original genus. They retrieved the Gammaproteobacteria group as the most represented among protease-producing bacteria isolated from marine sediments, mainly dominated by *Pseudoalteromonas, Shewanella, Colwellia* and *Planococcus* members. Gesheva and Vasileva-Tonkova [179] focused on the influence of culture medium supplementation with specific substrates on the enzymatic activities of microbial isolates from Antarctic soils. It was observed that while proteolytic and lipolytic activities of *Nocardioides* sp. strain were not affected by the carbon sources in the medium, amylase was favoured when wheat bran and soy-bean were added to the medium, while RNase activity was absent in cell-free supernatants obtained by culture with addition of sunflower oil, waste frying oil, kerosene and phenanthrene.

A dual approach combining culture-based and metagenomic techniques was applied on samples of the ikaite columns of SW Greenland to achieve the discovery of novel enzymes. The approaches demonstrated the presence of cold and/or alkaline-active enzymes and strengthened the complementarity between the two approaches. If on the one hand the investigations showed high hit-rate but also a strong phylogenetic redundancy, on the other the metagenomic analysis revealed a higher degree of phylogenetic novelty but also a lower hit-rates and low expression levels in the enzymatic activities. The β-galactosidase BGal_I17E2_ was suggested as a suitable compound for application in the dairy industry because it is able to hydrolyse lactose at low temperature [180].

Noteworthy, the study by Rizzo et al. [66] represents the first attempt to bioprospect bacterial communities associated to peculiar Antarctic brine habitats, evidencing great potentials in enzyme production. Interestingly, the isolates showed amylase, lipase/esterase, gelatinase, chitinase, DNase and haemolytic activity at low temperature (especially among *Pseudomonas* isolates) by supporting the role of these communities in the mineralisation of organic matter in briny ecosystems.

The enzymatic abilities of bacteria from Arctic environments have been less investigated than the Antarctic resources, but presented an equal potential in terms of relevant molecules with enzymatic functions. For instance, the 48% and 70% of strains isolated from the Wijdefjorden (Svalbard, Spitsbergen) and screened by Konieczna et al. [181] revealed respectively ureolytic and proteolytic activity. The same enzymatic activities with different proportion (32% and 55% for ureolytic and proteolytic, respectively) were instead detected for bacteria isolated from freshwater samples in the same area. *Pseudoalteromonas* was the genus most frequently ascertained among positive isolates, in line with other results obtained for the opposite pole. Similar percentages of positive strains were proved by De Santi et al. [13] for bacterial isolates from deep sediments, seawater and biota (animals and algae, see below) in the Lofoten area (Northern Norway) and on the coastal areas around the Svalbard archipelago. Indeed, esterase/lipase, DNase and protease were detected in more than 50% of screened strains, while amylase, chitinase and xylanase were reported for a 41, 23, 9 and 7% of the total strains. The isolation of possible new bacterial species was evidenced, and the enzymatic activity was mainly attributed to Gram negative bacteria, with some activities (tributyrin, skim milk and DNA degradation) that were equally distributed among Proteobacteria, Bacteroidetes, Actinobacteria and Firmicutes members. A total of 103 bacterial isolates from Ny-Ǻlesund soil samples, (Svalbard, Arctic) were investigated for enzymatic abilities, and among 47 phylotypes detected (belonging to the phyla Actinobacteria, Bacilli, Bacteroidetes and Proteobacteria) 26 phylotypes showed amylase and lipase activity at 5 °C and/or 20 °C, while no protease activity was detected [134]. Groudieva et al. [182] used different matrices to isolate cold-adapted strain to screen for enzymatic activities. Indeed, a total of 116 strains were isolated from sea ice samples of four permanently cold fjords of Spitzbergen, Arctic Ocean, and analysis on their enzymatic activities showed a wide variety of enzymatic activities, being able to degrade several kinds of proteins, lipids and polysaccharides with higher percentages for proteolytic activity. Interestingly, the authors revealed the unique feature to work at temperature as the water freezing point for α-amylase and β-galactosidase. All these findings are of great concern to elucidate the decomposition processes of biopolymers in the sea ice and underlying seawater which are still poorly understood. Among the isolates screened by Gratia et al. [166] (above discussed for the Antarctic area), the strain the strain *Arthrobacter psychrolactophilus* Sp 31.3 isolated from sand of a freshwater pond samples was selected as most promising for its ability to grow and to produce exoenzymes at low temperatures.

#### 2.4.2. Cold-Enzyme Producers from Biotic Matrices

Even in the case of investigations about cold enzymes, the use of biotic matrices from polar environments is still scantly improved. Only some reports are available about the use of living organisms or part of them to isolate bacterial enzyme producers, but no studies directly aimed at examining the potential of biotic matrices to isolate this bacterial fraction are available. Rentier-Delrue et al. [183] (1993) cloned and determined the triosephosphate isomerase gene from the Antarctic bacterium *Moraxella* TA 137 isolated from the intestine of an Antarctic fish, focusing on the temperature adaptation of the catalytic activity. The authors showed a strong dependence of enzymatic activities on the incubation temperature.

Loperena et al. [174] used bird remains, algae, bryophyte and microbial mat samples as source of isolation of enzyme bacterial producers, in addition to other abiotic sources. The highest lipase activity was detected for *Psychrobacter*, *Pseudomonas* and *Arthrobacter* members, originating from krill remains and bryophyte samples.

More recently, Herrera and coauthors [184] hypothesised that the oligochaete *Grania* sp. possess a specific microbiota with enzyme-producing ability, useful for the worm’s nutrient uptake. The study revealed thirty-four associated microorganisms able to produce different enzymes, including extracellular proteases, esterases, amylases, cellulases and agarases. Interestingly, the oligochaete is used to feed on debris of marine algae, thus the authors suggested a possible symbiotic relationship in which the associated microbiota assist *Grania* sp. in the recovery of nutrients deriving from algal biomass. *Flavobacterium*, *Pseudomonas*, *Salinibacterium* and *Psychrobacter* were the taxonomic groups to which enzyme producers resulted affiliated.

In the context of enzymatic processes, the hydrolysis of synthetic polymers is considered a burning issue, for which marine resources and cold environments are addressed with growing interest. Exciting results have been reported from a *Pseudomonas pelagia* strain isolated from a culture of the Antarctic green alga *Pyramimonas gelidicola*. [185]. Haernvall et al. [186] demonstrated the potential of this *P. pertucinogena* lineage member to hydrolyse ionic phthalic acid-based polyesters, attributed to the putatively secreted lipase PpelaLip. On the base of these findings, bioinformatic tools have been subsequently applied to explore the catalytic and biosynthetic potential, by detecting the presence of polyester hydrolases, halohydrin dehalogenases, ω-transaminases, flavin-binding fluorescent proteins, polyhydroxyalkanoates and ectoin synthesis clusters [187].

As far as the Arctic area is concerned, we note the work of De Santi et al. [13] previously treated in which part of the bacteria with enzymatic activity had been isolated from marine microbiota (animals and algae).

## 3. Biodiversity and Ecological Role in Cold Environments

**Figure 1:**
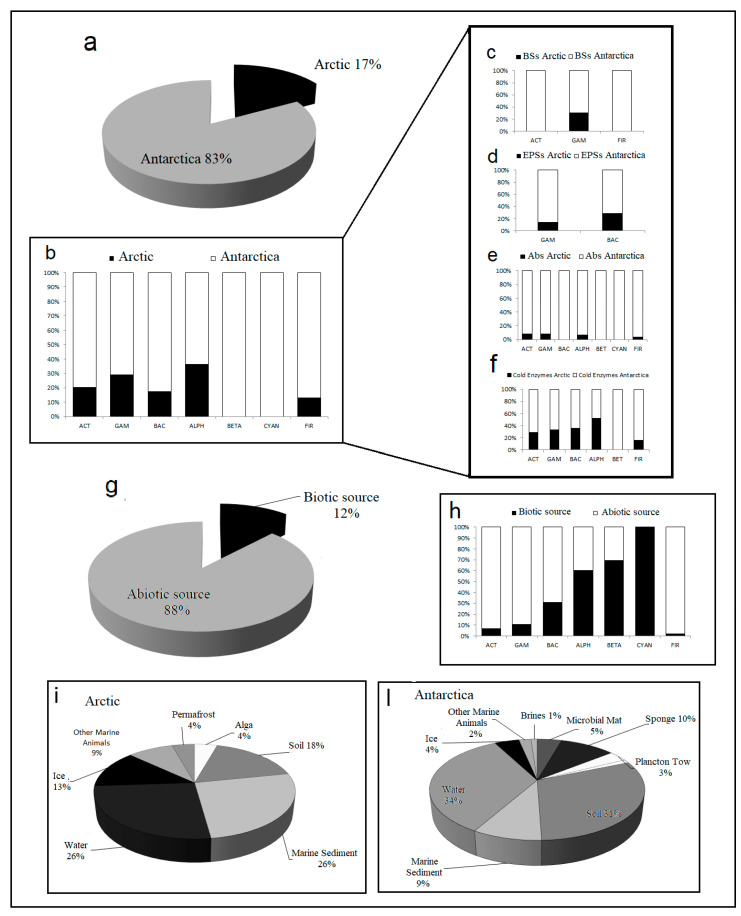
Exploration level of Arctic and Antarctic sources in bioprospecting field. (**a**) Number of studies exploring cold-adapted bacterial producers of biotechnological relevant molecules; (**b**) taxonomic groups detected as producers of biotechnological relevant molecules; (**c**–**f**) taxonomic groups detected as producers of biosurfactants (BSs), extracellular polymeric substances (EPSs), antibiotics (Abs) and cold-enzymes; (**g**) exploitation level of biotic and abiotic matrices used as source for isolation of bacterial producers; (**h**) taxonomic groups detected as producers of biotechnological relevant molecules from biotic and abiotic sources; (**i**,**l**) biotic and abiotic matrices explored in Arctic and Antarctica.

Figure 1 gives an idea about the exploitation level acting on both polar areas, Arctic and Antarctic. By considering all the bioactive molecules without distinction, Antarctica resulted the most explored source, even if literature is really poor. The 83% of references are about the use of Antarctic matrices as a source of new bioactive producers, while only the 17% are the attempts in the Arctic area (Figure 1a). The paucity with which this research topic has been investigated in the polar environment makes the data on the biodiversity of cold-adapted microorganisms as biomolecule producers very few and inconsistent to be able to assess broad-spectrum evaluations. However, it remains possible to make some interesting observations. All taxa resulted represented with higher percentages in Antarctic samples, but it is due to the higher number of references related to this area (Figure 1b–f). In the Antarctic samples, the most represented taxonomic groups reported as producers of an interesting compounds were Gammaproteobacteria (53.4%) and Actinobacteria (22.6%) members, as well as for Arctic environment with different values (67.2% and 17.6% for Gammaproteobacteria and Actinobacteria respectively). The other taxa have been also represented, but with lower percentages of isolates.

The strong representation of Actinobacteria is not a surprising data. Indeed, the phylum is well known as important pool from which to draw biologically active compounds [188], despite the massive focusing on this taxon led to a decreasing rate of isolation of new compounds was observed during past years. Among Actinobacteria members, bacteria reported were mainly affiliated to *Rhodococcus*, *Arthrobacter*, *Janobacterium*, *Brevibacterium*, *Kocuria*, *Lapillococcus*, *Leifsonia*, *Nesterenkonia*, *Terrabacter*, *Micromonospora*, *Streptomyces*, *Coryneform*, *Nocardioform*. Great part of these strains have been reported as producers of compounds with antimicrobial activity exhibited against different pathogens, including human pathogens and Bcc strains [189]. Gammaproteobacteria are instead predominated from the genera *Pseudoalteromonas* and *Psychrobacter*. The first genus was deeply studied and is considered a model study due to the frequency at which it is found in polar areas, thus is interesting for the improvement of cold adaptation [190,191,192]. Moreover, *Pseudoalteromonas* members have been reported for the presence of a high number of operons, often involved in the production of antimicrobial compounds [193]. More interestingly, together with these well-known genera, others have been detected less frequently among the bioactive molecules producers, thus confirming the possibility of identifying an ever-increasing number of new chemical structures. This is the case of *Colwellia*, *Shewanella*, *Pantoea*, *Idiomarina* and *Halomonas* members.

Arctic bioactive molecules producers were mainly affiliated to Gammaproteobacteria with an amount of 70% on the total of references, but interestingly in the Arctic area Bacteroidetes and Alphaproteobacteria members appeared well represented despite the paucity of available works. Specifically, *Polaribacter*, *Flavobacterium* and *Winogradskyella* have been reported in few and unique cases from both polar areas as innovative genera involved in BM biosynthesis, mainly focused on EPS production. Bioactivity rates of Firmicutes and Cyanobacteria members have been occasionally reported and only from Antarctica, mainly for antimicrobial compounds production from strains affiliated to different genera, namely *Bacillus*, *Planococcus*, *Sporosarcina*, *Enterococcus* and *Nostoc*, *Leptolyngbya*, *Phormidium*, respectively [189].

As regards to the matrices used, i.e., biotic and abiotic, the abiotic ones showed the absolute dominance, with a very low exploitation of biotic samples. Currently only the 12% (Figure 1g) of references reveal the use of biotic matrices for the isolation of bacterial strains differently distributed taxonomically (Figure 1h). The reported strains were able to produce biosurfactants, exopolysaccharides, antimicrobials or cold enzymes and were isolated from different biotic samples (i.e., sponges, microbial mat, algae) (Figure 1i,l). In Antarctica, the most used source of isolation among the abiotic matrices is represented by soil samples, while sponges represent at the moment the most used biotic source. In the Arctic, sediment and water samples are confirmed as most used samples, while the only biotic matrix is represented by a brown seaweed (Figure 1i,l). This widespread choice could be explained equally from scientific and technical assumptions. From a scientific point of view, researchers probably started to observe mainly soils because they are enriched with organic material during the summer season, thus supporting easily the development of microbial communities with abundance of nitrogen and phosphorous nutrients. On the other hand, in a more logistic vision the choice of soil samples could be dictated from the major accessibility to the sampling sites, or simplicity of processing operations.

This is true in general also for other abiotic sources of isolation of bacterial producers, which are less demanding in terms of timing, storage, procedures and materials for processing, while biotic samples face a whole series of care measures both during sampling and processing steps. This aspect probably represented a limit during the initial approach to bioprospecting in the polar areas which strongly affected the investigation of living organisms as suitable source of isolation. The latter has been considered only in recent time and for different reasons, strongly correlated to the occurrence of symbiosis relationships between bacteria and animal or vegetal organisms. Rizzo et al. [19,20] proposed the use of marine invertebrates as isolation sources of mesophilic bacterial BS-producers and reported polychaetes and sponges as optimal matrixes for the purpose. As regards to polar environments, the most used organisms are represented by sponges, and positive results have been obtained. Sponges are the best studied organisms in relation to symbiotic relationships with bacterial cells. The bacterial communities associated to Porifera are really complex and greatly specialised, characterised by an ecological equilibrium between competitive and cooperative interactions. As it was suggested by several authors, a complexively advantageous symbiosis requests the production of several metabolites for the complex cells-to-cells communication strategies within the bacterial assemblages. All these conditions have been proposed and scientifically supported for mesophilic environments and confirmed with some contributes also for polar invertebrates [71,72,194,195]. Therefore, the use of such organisms or other marine invertebrates is an intriguing opportunity of investigation for the discovery of new producers, or new natural chemical compounds.

## 4. Methodological Approach on Polar Environments for Bioprospectors

The advent of modern omics technologies has profoundly changed the way of doing research, especially in the ecological sector, and also in the field of microbial ecology and bioprospecting. Indeed, they constitute a significant scientific contribution for the comprehension of microbial adaptation strategies, also regarding cold-adapted microorganisms. The study of psychrophilic genomes and metagenomes pointed out a great capacity of microbial genomes to rearrange themselves in dependence of the requirements, through the acquisition of external genetic material or change within the own genetic equipment [2]. These genetic expression regulation strategies are translated in specialisations at molecular and structural modifications, from development of proteins and enzymes highly flexible and specific to more fluid cellular membrane and cytoplasmatic fluid enriched with antifreeze proteins and several cryoprotectant molecules.

A prove of that is the characterisation of chaperonin Cpn60 and co-chaperonin Cpn10 from an Antarctic seawater *Oleispira antarctica* strain RB-8 T, with high protein refolding activities in vitro at temperatures of 4–12 °C. The genes encoding the two chaperonins (*cpn*60 and *cpn*10) were cloned and expressed in *E.coli* to test the possibility of a growth range extension at lower temperatures, with successful results which led to the commercialisation of the engineered *E. coli* strain as ‘Arctic Express’ [196].

The genomic approach in studying biomolecules could be a useful tool also for the interpretation of metagenomics data. The functional metagenomics is currently revealed as the fastest and most accurate research key to increase the chances of identifying new biomolecules, especially in extreme environments, in which resides the most conspicuous fraction of not yet cultivable bacteria. Recent reports have applied such approach to investigate the Antarctic sponge-associated communities and to predict their functional role. Both Steinert et al. [197] and Papale et al. [198] studied the bacterial communities associated with Antarctic sponges (from two different areas) by carrying out a predictive analysis on metagenome. The authors detected a possible involvement of associated bacteria in xenobiotic biodegradation and secondary metabolites biosynthesis.

The progress of omics technologies also assumes great importance because of the enormous amount of data it makes available to the scientific community. The existence of databases in which an increasing number of bacterial genomic sequences are deposited represents a precious resource for all those who want to approach various fields of research. In the specific case of biosprospecting, the use and interpretation of previous and available data can be used to better target analysis or to detect microdiversity within close phylogenetical groups, or gene clusters encoding bioactive molecules [2].

Despite the undeniable progresses, the molecular approach could not be enough for an exhaustive analysis applicable to bioprospecting field. The collection of new isolates remains one of the most important starting base, in order to improve on the one hand, the knowledge of genomic sequences and information, and on the other hand the phenotype and the physiology of cold-adapted bacteria. To achieve these purposes, it is necessary to develop new and more sophisticated cultivation methods and more efficient collection procedure which could get access to new habitats, including the harsher ones. Certainly, new expeditions to the poles, and in particular to the most peculiar polar sites, are increasingly useful, with the aim to isolate new potential bacterial producers, and to analyse the microbial genomic pools with a more focused perspectives, in order to get closer to what is still undiscovered. But, Perfumo et al. [2] assumed important considerations in relation to this topic. Indeed, even if new expeditions are necessary, a lot of material useful for further research is just currently available also in the culturable world. As an example, several bacterial collection of isolates entirely dedicated to strains isolated from polar regions are accessible, and sometimes together with useful information about their biosynthesis ability or genomic heritages of these bacteria [106,199,200].

## 5. Conclusions

Bioprospecting, and polar environments as a source of investigation, is one of the most stimulating branches that currently feed the research. In a first attempt, one could think that cold-adapted organisms, and microorganisms in particular, possess a slow metabolic rate, and consider them a non-optimal resource for application in industrial processes for this reason. But the perspectives from which the topic should be analysed are different, and finally it is now emerging. Indeed, psychrophiles are adapted to be efficient, to adjust their biosynthesis rates in dependence on the environmental conditions and on their needs. The result is that they are able to maximise the yield by preserving energy, by reaching optimal performance at lowest temperature than those at which growth is fastest [201]. The research interest for relevant molecules in biotechnological field has existed for a long time, but it was focused mainly on terrestrial environments. However, what it has already been discovered and obtained is not in vain but can be applied to cold-adapted bacteria to verify differences in efficiency and convenience. As evidenced in this review, many more studies were focused on antibiotic and cold-enzyme production, while the biosurfactant production topic is seriously poorly improved. Moreover, really often the results show an overlap in the areas, because each biomolecule can cover more than one function, such as the case of biosurfactants which also perform antimicrobial action. The polar areas have been still little explored, and explored in different extent. Arctic is currently less explored, despite its resources are not legally protected as are the Antarctic ones. In addition, in both Poles biotic sources have been scarcely considered, but now is clear their huge potential. Clearly, this text is not intended as an invitation to the race for sampling, but was aimed at highlighting the importance of some resources existing on the earth as unique and peculiar. The few researches carried out and reported here have shown very encouraging results, which however must be completed and deepened to have a less fragmented and more exhaustive literature. Underlining the importance of polar resources, and in particular of biotic and genetic ones, is for the authors an equivalent to supporting the importance of their protection and safeguarding. The use of microorganisms as final producer of relevant biomolecules is also a suggestion in line with this assumption. Indeed, they could solve the problem correlated to the massive sampling of superior higher animals, mainly invertebrates, which for a long time have been considered the direct source of bioactive molecules. If polar bioprospecting could be the challenge of the future, there is a need for a comprehensive bioprospecting policy. This should be based on clearer and specific rules, starting from the scientific designs and the collection procedures, auspicable through national and international discussions aimed at ensuring regulation across all sectors, by preventing biopiracy episodes and over pressure on environments.

## Figures and Tables

**Table 1:** List of cold-adapted biosurfactant (BS) bacterial producers considered for the review paper.

Origin	Strain	Chemical Elucidation	Reference
**Antarctic_Abiotic Sources**			
Seawater	*Oceanobacillus* sp. BRI 10	Glycoproteic BS	[42,47]
Sea-ice/seawater interface (Terra Nova Bay)	*Halomonas* sp. ANT-3b	Glycolipidic BS	[40]
Seawater (Terra Nova Bay)	*Rhodococcus* sp.	Trehalose lipids	[43]
Seawater (Terra Nova Bay)	*Rhodococcus* sp.	Trehalose lipids	[43]
Seawater (Terra Nova Bay)	*Rhodococcus* sp.		[44]
Antarctic lakes, Cotton Glacier	*Janthinobacterium* sp., *Serratia* sp., *Psychrobacter* sp.	Sophorolipids and Di-Rhamnolipids	[45]
Sediment (Terra Nova Bay)	*Pseudomonas* sp. BNT1	Rhamnolipids	[46]
Sand (Deception Island)	*Bacillus licheniformis* AL 1.1	Lipopeptide	[36]
Soil (Peninsula Byers, Fildes Bay, Robert Island, Doumer Island, Fildes Bay-Escudero Base)	*Streptomyces luridus So3.2*	nd°	[18]
Soil (King George Island)	*Bacillus* spp., *Sporosarcina* spp., *Paenibacillus antarticus*	nd°	[38]
Soil (Casey Station)	*Rhodococcus fascians* A3	Rhamnolipids	[39]
Soil (Casey Station, Dewart Island, Terra Nova Bay)	*Coryneform* sp. A1, A3, A9, A11, A14, A16 *Nocardioform sp.* A8, A15, A17 *Micromonospora* sp. A10	Glycolipids	[49]
Soil (Dewart Island)	*Pantoea* sp. strain A-13		[50]
Antarctic soil enrichments	*Idiomarina loihiensis* L2TR sp. 185	nd°	[35]
	*Pseudoalteromonas* BG-1-E1 sp.93	nd°	
	*Pseudomonas* sp. AC4 sp. 235	nd°	
	*Rhodococcus* spp. 174, 176, 179-181, 187, 188, 190-192, 224, 225, 227, 231	nd°	
Freshwater (Svaldbard Island)	*Pseudomonas fluorescens* BD5	Lipopeptides (Pseudofactin I, Pseudofactin II)	[51]
Soil (Svalbard Archipelago)	*Pseudomonas putida* BD2	Rhamnolipids	[14]
Arctic soil enrichments	*Pseudomonas* sp. 280	nd	[35]
**Arctic_Biotic sources**			
*Hippoglossus hippoglossus*	*Pseudomonas sp.* M10B774	Rhamnolipids	[29]

°nd, not determined.

**Table 2:** List of cold adapted extracellular polymeric substances (EPS) bacterial producers considered for the review paper.

Origin	Strain	Chemical Elucidation *	Sugar Content	Ref.
**Antarctic_Abiotic sources**				
Seawater (Terra Nova Bay)	*Pseudoalteromonas* sp. MER144	CRB, 18%; UA, 14%; PRT, 12%	Glc, Man, GalN, Ara, GlcA, GalA, Gal (1:0.36:0.26:0.06:0.06:0.05:0.03)	[55]
Seawater	*Pseudoalteromonas haloplanktis* TAC 125	ND	Man, Glc	[59]
Seawater (Terra Nova Bay)	*Marinobacter* sp. W1-16	CRB, 38%; UA, 2.7%; PRT, 7%	Glc, Man, Gal, GalN, GalA, GlcA (1:0.9:0.2:0.1:0.1:0.01)	[65]
Sediment (King George Island)	*Pseudoalteromonas* sp. KOPRI	ND	Gal, Glc (1:1.5)	[60]
Sediment (South Shetland Islands)	*Pseudomonas* sp. ID1	CRB, 33.81%; UA, 2.40%; PRT, 2.81%	Glc, Gal, Fuc	[58]
Melted fast ice Antarctic	*Flavobacterium* sp. CAM005	NS ≈ 50%; PRT ≈ 40%, AS and UA presence	Man, Glc, GlcA, Ara, Gal, GlcNAc	[61,62]
	*Shewanella* sp. CAM090	NS ≈ 40%; UA ≈ 40%; PRT ≈ 15%, AS presence	Man, GlcA, Ara, Glc, GlcNAc, Gal, Xyl, Rha	
	*Pseudoalteromonas* sp. CAM003	NS ≈ 50%; UA ≈ 10%; PRT ≈ 20%, SULF ≈ 20%; AS presence	Man, Fuc, Glc, Rha, Ara, Rib, GlcA, GalNAc, GlcNAc	
	*Pseudoalteromonas* sp. CAM015	NS ≈ 40%; UA ≈ 30%; PRT ≈ 30%	Glc, Man, Ara, Rha, Gal, GlcA, GalNAc, Xyl	
	*Pseudoalteromonas* sp. CAM064	NS ≈ 50%; UA ≈ 30%; PRT ≈ 10%, AS ≈ 10%, SUL presence	Man, GalNAc, Glc, GlcA, Ara, Gal, GlcNAc	
Particles from Antarctic sea	*Pseudoalteromonas* sp. CAM025	NS, 74%; UA, 22%; PRT, 2%, SUL, 5%	Glc, GalA, Gal, Rha, Ara, Fuc, Rib, Man, GalNAc	[62]
	*Pseudoalteromonas* sp. CAM036	NS, 50%; UA, 25%; PRT, 3%, SUL 5%	GalA, Glc, Man, GalNAc, Ara, Gal	
Seawater (Arctic Ocean)	*Pseudoalteromonas elyakovii* sp. ArcPo15	ND	Man, GalA (3.3:1.0)	[70]
Sediments	*Colwellia psychrerythraea* 34H	ND	ND	[69]
Glacier soil (Ny-Ålesund, Svalbard)	*Flavobacterium* sp. ASB 3-3	CRB, 56%; PRT, 23%; SUL, 21%	Glc, D-galactose	[67]
Sea-ice	*Pseudoalteromonas* sp.	ND	Man, Glc, Gal, GlcNAc, Rha, GalNAc, Xyl	[8]
**Antarctic_Biotic sources**				
Plankton tow	*Flavobacteriaceae* CAM030,	NS ≈ 40%; UA ≈ 30%; PRT ≈ 20%, AS ≈ 15%	Man, GlcA, Glc, GalNAc, Ara, Gal, GalA, GlcNAc, Xyl, Rha	[61,62]
	*Pseudoalteromonas* sp. CAM023	NS ≈ 70%; UA ≈ 20%; PRT ≈ 10%, AS presence	Glc, Ara, GalA, GlcA, GalNAc, Man, Gal	
	*Polaribacter* sp. CAM006	NS ≈ 30%; PRT ≈ 45%, AS and UA presence	Gal, Man, Fuc, GlcA, Glc, GlcNAc, Ara, GalNAc,	
Antarctic sponges	*Colwellia* sp. GW185	CRB, 28%; PRT, 2.08%; UA, 6.09%	Glc, Man, Gal, GalN, GlcA, GalA (1:1:0.7:0.7:0.3:0.04)	[71]
Antarctic sponges	*Shewanella* sp. CAL606	CRB, 26%; PRT, 3%; UA, 6.07%	Glc, Gal, Man, GalN, GlcA, GalA (1:1:0.9:0.6:0.3:0.1)	
Antarctic sponges	*Winogradskyella* sp. CAL396	CRB, 21%; PRT, 8.8%; SUL, 3.2%	Man, Ara, GalA, GlcA, Gal, Glc, GlcN (1:0.9:0.4:0.3:0.2:0.2:0.01)	
Antarctic sponges	*Winogradskyella* sp. CAL384	CRB, 15%; PRT, 2.4%; UA, 11.9%	Glc, Man, GalA, Ara, Gal, GlcN, GlcA (1:0.5:0.3:0.25:0.1:0.1:0.1)	
**Arctic_Biotic sources**				
Brown alga	*Polaribacter* sp. SM1127	ND	GlcNAc, Man, GlcA, Gal, Fuc, Glc, Rha	[15]

* CRB, carbohydrates; UA, uronic acids; PRT, proteins; SUL, sulphates; NS, neutral sugar; ND, not determined. Glc, glucose; Man, mannose; GalN; Ara, arabinose; GlcA, glucuronic acid; GalA, galacturonic A; Gal, galactose; Fuc, fucose; GlcNAc, N-Acetyl-D-glucosamine; Xyl, xylose; Rha, rhamnose; GalNAc, N-Acetyl-D-galactosamine; GlcN, glucosamine.

**Table 3:** List of cold adapted Abs bacterial producers considered for the review paper.

Origin	Taxonomic Affiliation	Target	Reference
**Antarctic_Abiotic sources**			
Soils (Cape Hallett, Edmonson Point, Kay Island, Cape Russell, Lake Hoare, Harrow Peaks, Crater Circe, Battleship Promontory, Mount, McGee, Mount Rittmann, Mount Melbourne)	*Arthrobacter*, *Planococcus*, *Pseudomonas*	*L. innocua*, *P fragi*, *B. thermosphacta*, *S. aureus*, *L. monocytogenes*	[74]
Soils (Deception Island, Shetland Islands, Galindez Island, Argentine Islands)	*Arthrobacter*, *Sporosarcina*, *Bacillus*, *Pseudomonas*, *Burkholderia*, *Rhodococcus*, *Janthinobacterium*	*E. coli*, *P. Aeruginosa, A. johnsonii*	[101]
Soils (Penguin rookeries Larsemann Hills)	*Enterococcus, Psychrobacter, Bacillus*	*Candida albicans*	[85]
Soils (Fildes Peninsula, King George Island)	*Janthinobacterium* sp. SMN 33.6	*S. marcescens*, *E. coli*, *A. baumannii, P. aeruginosa*	[89]
Soils	*Streptomyces griseus*	*B. subtilis, S. aureus*	[102]
Soils	*Streptomyces*	*B. subtlis, C. michiganensis, B. cepcia, B. pyrrocinia, B. gladioli, E. amylowora, E. coli*	[103]
Soils	*Streptomyces* INACH3013	*S. aureus*	[104]
Soil (Deception Island)	*Gordonia terrae, Leifsonia* *Terrabacter*	*Salmonella paratyphi A, Salmonella typhimurium*	[105]
Soils (Barrientos Island)	*Brevibacterium*, *Janibacter*, *Kocuria*, *Demetria*, *Gordonia*, *Lapillicoccus*, *Micromonospora*, *Nocardioides* sp., *Rhodococcus* sp.	*C. albicans*, *S. aureus*, *P. aeruginosa*	[106]
Soils (Terranova Bay)	*Pseudomonas*	*Burkholderia cepacia* complex	[46]
Soils (Casey Station, Wilkes Land)	*Nocardioides*	*S. aureus*, *X. oryza*	[179]
Soils (King George Island)	*Pedobacter*, *Pseudomonas*	*E. coli*, *Salmonella* spp., *K. pneumoniae*, *E. cloacae*, *V. parahaemolyticus*, *B. cereus*	[109]
Soils (King George Island)	*Sporosarcina*, *Bacillus*	*S. aureus*, *C. albicans*	[38]
Sediments (Deception Island, Martel Bay, King George Island, Punta Hannah	*Pseudomonas, Bacillus, Marinobacter, Sulfitobacter, Flavobacterium, Tsukamurella, Cyclobacterium, Cellulophaga, Arthrobacter, Streptomyces, Pseudoalteromonas*	*E. coli*, *M. luteus*, *S. aureus*, *B. subtilis*, *C. albicans*	[110]
Freshwater, (Schirmacher Oasis)	*Janthinobacterium* sp. Ant5-2 *Flavobacterium* sp. Ant342	Virulent *Mycobacterium smegmatis,* Avirulent *Mycobacterium tuberculosis*	[107]
Freshwater lake, Skarvsnes region	*Lysobacter oligotrophicus*	*E. coli, L. enzymogenes, R. appendicifer, S. cerevisiae*	[157]
Seawater (Stations Mergellina Santa Maria, Novella, Tiburtina, Road Bay, Gerlache Inlet, Evans Cove, Inexpressible Island, Cape Hallet, Tethys Bay)	*Arthrobacter*, *Janibacter thuringensis, Rhodococcus fascians, Nesterenkonia*, *Pseudoalteromonas*	*P. aeruginosa*, *S. aureus*, *Salmonella enterica*, *C. albicans*	[76]
Seawater (Antarctic Peninsula, South Shetland Islands)	*Halomonas titanicae*	*E. coli*, *S. aureus*	[100]
Seawater	*Pseudoalteromonas haloplanktis* TAC125	*Burkholderia cepacia* complex	[72]
Seawater	*Pseudoalteromonas haloplanktis* TAC125	*S. epidermidis*	[113]
Seawater (King George Island)	*Pseudomonas fragi*	Antibiofilm *Flavobacterium psychrophilum*	[114]
Seawater	*Bacillus*	Antifungal *Paecilomyces variotii, Colletotrichum gloeosporioides, Fusarium oxysporum, Trichoderma viride, Rhizoctonia solani Kühn, Alternaria longipes, Sclerotinia sclerrotioru*	[108]
Seawater, French Antarctic station Dumont d’ Urville, Terre Adélie	*Pseudoalteromonas haloplanktis* TAC125	*Burkholderia cepacia* complex	[112]
Soils, water	*Pseudoalteromonas* sp. S8-8, S8-38, TAB23, TAE56, TAE79, TAE80, TAC125	*Burkholderia cepacia* complex	[194]
**Arctic_Abiotic sources**			
Sediments	*Paracoccus* sp. Arc7-R13	*B. subtilis*, *S. aureus*, *P. aeruginosa*, *E. coli*	[115]
Seawater (Chuckchi Sea)	*Pseudomonas aeruginosa*	*S. aureus, C. albicans*	[117]
Kongsfjorden (Svalbard Islands)	*Salinibacterium spp.* C3W3, C2W9	*P.damselae* subsp. *piscicida*	[118]
Seawater, sea ice	*Arthrobacter*, *Psychrobacter, Pseudoalteromonas, Vibrio*	*V. anguillarum*, *S. aureus*	[120]
Glacial melt water, sea convergence (Ny-Alesund)	*Yersinia aldovae, Carnobacterium maltaromaticum*	*Candida albicans*	[85]
Permafrost, saline spring sediments, and cryptoendoliths	*Paenibacillus* sp. GHS.8.NWYW.5 *Pseudomonas* sp. ALPS.10.MNAAK.13	*S. aureus, L. monocytogenes* *, S. enterica, E. coli* *, A. baumanii, E. faecium* *, E. faecalis*	[121]
**Antarctic_Biotic sources**			
Sponge *Isodictya setifera*	*Pseudomonas aeruginosa*		[122]
Benthic microbial mat (Larsemann Hills, Bølingen Islands, Vestfold Hills, Rauer Islands, the McMurdo Dry Valleys)	*Nostoc* CCC537	*M. tuberculosis, S. aureus, Salmonella typhi, P. aeruginosa, E coli, E. aerogenes*	[123]
Benthic microbial mat (Larsemann Hills, Vestfold Hills, McMurdo Dry Valleys)	*Psychrobacter*, *Shewanella*, *Arthrobacter*, *Janthinobacterium, Flavobacterium, Hymenobacter, Microbacterium, Micrococcus, Bacillus, Brevundimonas, Mesorhizobium, Pseudomonas, Hydrogenophaga, Marinobacter*	*S. aureus, Enterococcus faecium, E. coli* *Cryptococcus neoformans, Aspergillus fumigatus, C. albicans*	[124]
Benthic microbial mat (Larsemann Hills, Bølingen Islands, Vestfold Hills, Rauer Islands, the McMurdo Dry Valleys)	*Leptolyngbya antartica, Phormidium priestleyi, Phormidium murrayi, Nostoc*	*S. aureus* Antifungal *A. fumigatus, C. neoformans*	[125]
Sponges *Haliclonissa verrucosa, Anoxycalyx joubini, Lissodendoryx nobilis*	*Pseudoalteromonas haloplanktis* TB41, *Psychrobacter* sp. TB67, TB47, *Arthrobacter* sp. TB23	*Burkholderia cepacia* complex	[72,111]
Antarctic sponges	*Pseudoalteromonas* sp. TB13, TB25, TB41, TB51, TB64	*Burkholderia cepacia* complex	[194]
Antarctic sponges	*Shewanella* sp. TB4	*Burkholderia cepacia* complex	[195]
Antarctic sponges	*Pseudoalteromonas* sp. AC163	*Burkholderia cepacia* complex	[193]

**Table 4:** List of cold-enzymes bacterial producers considered for the review paper.

Origin	Taxonomic Affiliation	Chemical elucidation	Reference
**Antarctic_Abiotic sources**			
Seawater (Dumont d’Urville)	*Moraxella* *TAI44*	Lipases	[153]
Seawater (Dumont d’Urville)	*AIteromonas haloplanctis A23*	α-Amylase	[147,148,149,150]
Seawater (Dumont d’Urville)	*Bacillus* sp. TA39	Subtilisin	[151]
Seawater (Dumont d’Urville)	*Bacillus* sp. TA41	Subtilisin	[152]
Seawater (Dumont d’Urville)	*Psychrobacter immobilis* BI0	Lipases	[155,156]
Freshwater lake	*Lysobacter oligotrophicus*	Esterase, Amylase, Protease	[157]
Freshwater lake (Lake Yukidori Ike, Lake Hotoke Ike, Lake Skallen Oike)	*Flavobacterium, Pseudomonas, Arthrobacter, Psychrobacter, Cryobacterium, Hymenobacter, Polaromonas*	Protease (metalloproteases)	[160]
	*P. cryohalolentis strain ANH4-1*	Protease (serine protease)	
Seawater, freshwater, soils, sediments, remains of organic matter	*Pseudomonas*, *Pseudoalteromonas*	Protease	[133]
Seawater (King George Island)	*Pseudoalteromonas* sp. strain P96-47	Protease (metalloproteases)	[161]
Sea ice	*Colwellia* sp. NJ341	Protease (serine protease)	[163]
Sea ice	*Marinomonas* sp. NJ522	Superoxide dismutase	[165]
Antarctic soils (King George Island)	*Sporosarcina aquimarina, Algoriphagus antarcticus*	Protease	[159]
Soils	*Bacillus* sp. JSP1	Protease	[162]
Soils (Casey Station, Wilkes Land)	*Nocardioides* A-1	Protease, Amylase, Lipase, RNAse, Phosphatases, Ureases, Cellulase	[179]
Soils (King George Island)	*Bacillus, Sporosarcina, Paenibacillus, Rummeliibacillus*	Proteases, Amylase, Cellulase, Esterase, Lipase, Chitinase, Gelatinase	[38]
Sediments and soils (Deception Island, Galindez Island)	*Arthrobacter*, *Rhodococcus*, *Bacillus*, *Sporosarcina*, *Pseudomonas*, *Janthinobacterium*, *Burkholderia*	Poly-enzymatic activity (Ureases, polygalacturonases, β-glucosidases, phytases, ribonucleases, polygalacturonase)	[164]
Not specified (Terre Adelie, Deep sea samples)	*Pseudomonas, Psychrobacter, Flavobacterium, Rhodococcus, Arthrobacter, Sporosarcina, Planococcus, Kocuria*	Poly-enzymatic activity (Proteases, Lipases, Amylases, Cellulases and Xylanases)	[166]
Deep-sea sediments (Southern Okinawa Trough)	*Halomonas*, *Psychrobacter*	Poly-enzymatic activity (Amylases, Proteases, Lipases, Dnases	[172]
Sediments	*Pseudoalteromonas, Shewanella, Colwellia*, *Planococcus,*	Proteases, thermokinesis	[178]
Water, soils (Potter Cove)	*Pseudoalteromona*, *Pseudomonas, Flavobacterium, Olleya, Psychrobacter*, *Psychromonas*, *Colwellia*, *Shewanella, Polaribacter, Planococcus, Kocuria, Hydrrogenophaga, Arthrobacter, Salinibacterium, Planomicrobium, Lacinutrix, Cellulophaga,*	Poly-enzymatic activity (Proteases, Pectinases, Cellulases, Xylanases, Amylases, Agarases)	[135]
Air, ice, sea and freshwater, soil, sediment, bird and marine animal faeces, dead animals, rocks	*Pseudomonas*, *Psychrobacter, Arthrobacte*, *Bacillus, Carnobacterium*	Poly-enzymatic activity (Amylase, Lipase, Gelatinase, Caseinase, Protease, Ligninase, Xylanase, Cellulase)	[174]
Soils, marine and lake sediment, sea water (South Shetland Islands)	*Arthrobacter, Brevibacterium, Curtobacterium, Janibacter, Knoellia, Rhodococcus, Streptomyces, Thermoleophilum*	Protease, Gelatinase, Cellulase	[175]
Antarctic brines (Boulder Clay Lake)	*Pseudomonas, Psychrobacter, Shewanella, Gelidibacter, Staphylococcus, Carnobacterium, Rhodobacter, Leifsonia, Devosia, Sporosarcina, Marinobacter, Cryobacterium, Rothia, Rhodoglobus*	Oxidase, Catalase, Amylase, Lipase/Esterase, Gelatinase, Chitinase, DNase, Haemolytic activity	[66]
**Arctic_Abiotic sources**			
Sediments (Kongsfjorden), sediments, soils sample from Ny-Ålesund, Svalbard)	*Brevundimonas, Paracoccus, Roseovarius, Sphingomonas, Sphingopyxis, Sulfitobacter, Acinetobacter, Colwellia, Enhydrobacter, Marinobacter, Marinomonas, Marinobacterium, Oceanisphaer, Photobacterium, Pseudomonas, Pseudoalteromonas, Psychrobacter, Shewaella, Flavibacterium, Lacinutrix, Maribacter, Winogradskyella, Zoobellia, Cyclobacterium, Arthrobacter, Rhodococcus, Salinibacterium, Planococcus*	Amilase, Lipase	[134]
Sand of a freshwater pond (Ny-Alesund Arctic)	*Arthrobacter psychrolactophilus Sp 31.3*	Poly-enzymatic activity (Proteases, Lipases, Amylases, Cellulases and Xylanases)	[166]
Sediments, Freshwater (Wijdefjorden and Woodfjorden, Spitsbergen)	*Pseudomonas, Pseudoalteromonas*	Urease, Protease	[181]
Sediments, seawater (Lofoten area, NorthernNorway)	*Arthrobacter, Clavibacter, Filibacter, Leifsonia, Planococcus, Rhodococcus, Streptomyce, Flavobacterium, Gelidibacter, Marinobacter, Nesterenkonia, Nocardiopsis, Micrococcus*, *Planococcus, Plantibacter, Pseudoalteromonas, Pseudomonas, Psychromonas, Psychrobacillus, Halomonas, Marinomonas, Microbacterium, Rhodobacter, Roseobacter, Roseovarius, Serratia, Shewanella, Sporosarcina, Salinibacterium, Thalassospira*, *Streptomyces*, *Sanguibacter*, *Tomitella*, *Staphilococcus*, *Achromobacter*, *Acinetobacter*, *Brevundimonas*, *Bizonia*, *Hoeflea*, *Oceanisphaera*, *Moritella*, *Photobacterium*, *Polaribacter*, *Promiconospora*, *Gemmobacter*, *Celeribacter*, *Tropicibacter*, *Serratia*, *Pseudoruegeria*, *Sphingopyxis*, *Thalasospira*, *Stenotrophomonas*, *Sulfitobacter*, *Vibrio*	Esterase/Lipase, DNase, Protease, Amylase, Chitinase, Xylanase	[13]
Sea ice (Spitzbergen, Arctic Ocean)	*Marinomonas, Colwellia, Psychromonas, Psychrobacter, Shewanella, Pseudomonas, Pseudoaltheromonas, Gelidibacter, Planomicrobium, Planococcus, Carnobacterium, Agreia, Arthrobacter, Rhodococcus, Brachybacterium*	Protease, Lipase, α-Amylase, β-galactosidase	[182]
**Antarctic_Biotic sources**			
Algae, bryophyte and microbial mat	*Pseudomonas*, *Psychrobacter, Arthrobacter*, *Bacillus*, *Carnobacterium, Thermoleophilum minutum*	Poly-enzimatic activity (Amylase, Lipase, Gelatinase, Caseinase, Protease, Ligninase, Xylanase, Cellulase)	[174]
Oligochaete *Grania* sp.	*Flavobacterium, Pseudomonas, Salinibacterium, Psychrobacter*	Proteases, Esterases, Amylases, Cellulases, Agarases	[184]
**Antarctic_Biotic sources**			
Green alga *Pyramimonas* *Gelidicola* culture	*Pseudomonas pelagia*	Polyester hydrolases	[185,186]
**Arctic_Biotic sources**			
Various microbiota (marine animals, algae)	*Arthrobacter, Clavibacter, Filibacter, Leifsonia, Planococcus, Rhodococcus, Streptomyce, Flavobacterium, Gelidibacter, Marinobacter, Nesterenkonia, Nocardiopsis, Micrococcus*, *Planococcus, Plantibacter, Pseudoalteromonas, Pseudomonas, Psychrobacter, Psychromonas, Psychrobacillus, Halomonas, Marinomonas, Microbacterium, Rhodobacter, Roseobacter, Roseovarius, Serratia, Shewanella, Sporosarcina, Salinibacterium, Thalassospira*, *Streptomyces*, *Sanguibacter*, *Tomitella*, *Staphilococcus*, *Achromobacter*, *Acinetobacter*, *Brevundimonas*, *Bizonia*, *Hoeflea*, *Oceanisphaera*, *Moritella*, *Photobacterium*, *Polaribacter*, *Promiconospora*, *Gemmobacter*, *Celeribacter*, *Tropicibacter*, *Serratia*, *Pseudoruegeria*, *Sphingopyxis*, *Thalasospira*, *Stenotrophomonas*, *Sulfitobacter*, *Vibrio*	Esterase/Lipase, DNase, Protease, Amylase, Chitinase, Xylanase	[13]
